# Obliteration of thyroid arteries as a new method of treatment of thyroid diseases

**DOI:** 10.1186/1756-6614-6-S2-A67

**Published:** 2013-04-05

**Authors:** Grzegorz Kamiński, Andrzej Jaroszuk

**Affiliations:** 1 Department of Endocrinology and Radioisotope Therapy, Military Institute of Medicine, Warsaw, Poland

## 

Standard methods including pharmacotherapy, radioiodine and surgery cannot always be applied in treatment of thyroid diseases. The limitations of these methods are: drug intolerance and their side effects, too low radioiodine uptake, high risk of surgery. The obliteration of thyroid arteries seems to be an alternative method which could be used in the stiuation of ineffective standard treatment. It is based on shut down of blood flow in chosen thyroid arteries by injection of embolizative material (histoacryl or particles of polyvinylic alcohol) into the vessels. The consequence of acute ischemia is a septic necrosis of the glandular tissue in a field being supplied by this particular artery. Further repair processes and fibrosis lead to the reduction of an active thyroid hormone synthesis and decrease of thyroid volume. Effects of the embolization on apoptosis induction and modulation of autoimmune reactions are also observed. Preoperative selective embolization of a huge goiter or thyroid cancer improves surgery outcomes, reduces the risk of haemorrhage and damage to surrounding tissue. Palliative use of embolization in advanced stages of thyroid cancer reduces symptoms and improves quality of life. Little invasive nature of this procedure in comparison to surgery, the lack of serious undesirable coincidence makes the embolization of thyroid arteries an attractive form of therapy, which may become a therapeutic option in many difficult clinical situations and may improve the effectiveness of treatment of thyroid disease. The authors present the experiences gained in their institution with conclusions as follows:

1. As a result of using embolization of thyroid arteries in the treatment of selected thyroid diseases the following observations have been made: a significant reduction in goiter volume with resolution of compression symptoms of adjacent organs, reduction of the concentration of thyroid antibodies characteristic for patients with Graves' disease (TRAb), cure of hyperthyroidism in 71% of patients with thyrotoxicosis. Embolization procedure was not associated with the occurrence of undesirable side effects, such as the clinical symptoms associated with increased concentration of free thyroid hormones, and induction or exacerbation of pre-existing autoimmune thyroid disease.

2. Thyroid artery embolization had no significant influence on activity of the parathyroid glands, regardless of the number and quality of closed vessels.

3. Thyroid artery embolization is an effective and safe treatment of selected thyroid diseases as an alternative to conventional forms of therapy.

